# Functional Fluorescent Ca^2+^ Indicator Proteins in Transgenic Mice under TET Control

**DOI:** 10.1371/journal.pbio.0020163

**Published:** 2004-06-15

**Authors:** Mazahir T Hasan, Rainer W Friedrich, Thomas Euler, Matthew E Larkum, Günter Giese, Matthias Both, Jens Duebel, Jack Waters, Hermann Bujard, Oliver Griesbeck, Roger Y Tsien, Takeharu Nagai, Atsushi Miyawaki, Winfried Denk

**Affiliations:** **1**Max Planck Institute for Medical ResearchHeidelbergGermany; **2**Universität HeidelbergZentrum für Molekulare Biologie HeidelbergGermany; **3**Department of Pharmacology and Howard Hughes Medical Institute, University of CaliforniaSan Diego, CaliforniaUnited States of America; **4**Brain Research Institute (RIKEN)SaitamaJapan

## Abstract

Genetically encoded fluorescent calcium indicator proteins (FCIPs) are promising tools to study calcium dynamics in many activity-dependent molecular and cellular processes. Great hopes—for the measurement of population activity, in particular—have therefore been placed on calcium indicators derived from the green fluorescent protein and their expression in (selected) neuronal populations. Calcium transients can rise within milliseconds, making them suitable as reporters of fast neuronal activity. We here report the production of stable transgenic mouse lines with two different functional calcium indicators, inverse pericam and camgaroo-2, under the control of the tetracycline-inducible promoter. Using a variety of in vitro and in vivo assays, we find that stimuli known to increase intracellular calcium concentration (somatically triggered action potentials (APs) and synaptic and sensory stimulation) can cause substantial and rapid changes in FCIP fluorescence of inverse pericam and camgaroo-2.

## Abstract 2

Winfred Denk and colleagues succeed in generating transgenic mice that express one of two calcium indicators in their cells, creating a valuable tool to study neuronal activity

## Introduction

Central to the study of neuronal networks is the simultaneous measurement of activity at many locations. While important results have been obtained using multiple patch recordings (Stuart et al. 1993; Markram 1997; Markram et al. 1997) and microelectrode arrays (Meister et al. 1994), patch recordings are limited to a few points and electrode arrays can only record spiking activity or compound field potentials. Furthermore, electrical recordings cannot resolve activity in fine branches of individual neurons and are blind to biochemical signals. Optophysiological approaches have, therefore, become strong competitors and complementors of valuable electrophysiological methods for studying neural activity. First attempts used intrinsic optical signals (Cohen et al. 1968) followed by specific chromophores for sensing membrane voltage by absorption (George et al. 1988) or fluorescence changes (for a review see Cohen et al. 1978). Other dyes were found (Gorman and Thomas 1978) and later specifically designed that respond to changes in intracellular calcium (Ca^2+^) concentration (for a review see Tsien 1992). Although changes in membrane potential are the most direct measurement of neuronal activity, the large fractional changes achievable with Ca^2+^-dependent fluorophores led to a rapid adoption of Ca^2+^ measurements (Tank et al. 1988; Ross et al. 1990; Sugimori and Llinas 1990), which acquired additional importance with the discovery that the induction of synaptic plasticity in many cases requires a substantial rise in local [Ca^2+^] (Malenka et al. 1988; Yang et al. 1999). Ca^2+^ furthermore plays a role in morphological changes of neurites (Yuste and Bonhoeffer 2001) and in gene regulation (Morgan and Curran 1986).

Loading a population of cells with Ca^2+^ indicators has proven difficult in adult neural tissues. While there has been a recent advance (Stosiek et al. 2003), it is unclear how cell-type specificity could ever be achieved by techniques of bulk loading synthetic indicators. Great excitement, therefore, greeted the molecular engineering, several years ago (Miyawaki et al. 1997; Persechini et al. 1997), of GFP variants that are Ca^2+^-sensitive (fluorescent calcium indicator proteins [FCIPs]). Two classes of genetic Ca^2+^ indicators have been designed that use different mechanisms of action. The first class, called “cameleons” (Miyawaki et al. 1997; Miyawaki et al. 1999; Nagai et al. 2002), depends on changes in the efficiency of fluorescence resonance energy transfer between two spectral variants of green fluorescent protein (GFP) that are connected by a Ca^2+^-sensitive linker. The second class uses a single GFP fluorophore that contains a Ca^2+^-dependent protein as a sequence insert (Baird et al. 1999; Griesbeck et al. 2001; Nagai et al. 2001). In addition to solving the loading problem, major advantages of genetic indicators are the prospect of targeting specific cell types by using appropriate promoters and the possibility of combining long-term studies of neuronal activity and morphology (Grutzendler et al. 2002; Trachtenberg et al. 2002).

The ability to express FCIPs in intact animals has in recent years allowed the measurement of [Ca^2+^] transients in worm (Kerr et al. 2000; Suzuki et al. 2003), fruitfly (Fiala et al. 2002; Reiff et al. 2002; Liu et al. 2003; Wang et al. 2003; Yu et al. 2003), zebrafish (Higashijima et al. 2003), and, more recently, mouse (Ji et al. 2004). Thus far, there are still no reports of transgenic mice that express functional FCIPs in the brain. Clearly, expression of a functional indicator in the mammalian brain would enable the measurement of neuronal population activity with much higher spatial and temporal resolution than are offered by currently used noninvasive methods, such as functional magnetic resonance imaging, positron emission tomography, and intrinsic signal reflectance imaging. Moreover, in combination with two-photon imaging (Denk et al. 1990; Denk and Svoboda 1997), transgenic indicators would allow the simultaneous recording of Ca^2+^ signals in neurons and neuronal compartments from multiple sites in vitro and in vivo.

In this paper we demonstrate that FCIPs can be transgenetically introduced into mice under the control of the tetracycline (TET) regulation system (for a review see Gossen and Bujard 2002) and are expressed and function widely throughout the nervous system.

## Results

### Construction of Transgenic Mice

**Figure 1:**
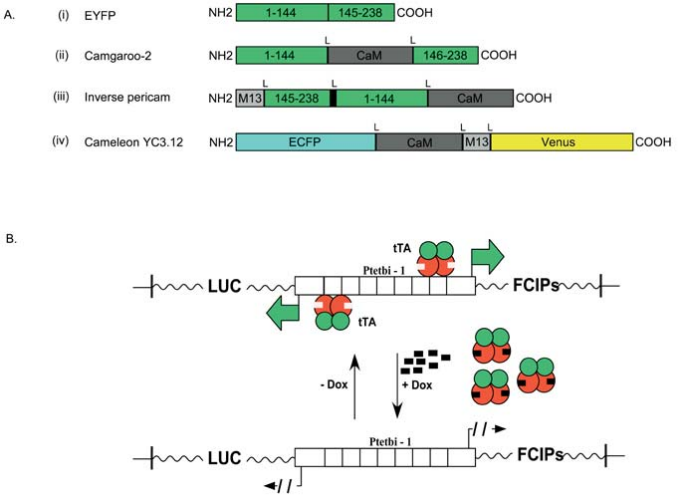
Genetic Designs of the FCIPs and the TET System (A) Genetic design of fluorescence Ca^2+^ indicator proteins: (i) yellow fluorescent protein, (ii) Cg2, (iii) IP, and (iv) came-leon YC3.12. (B) Operating principles of the TET regulatory system (for details see Gossen and Bujard 2002). “L” indicates short linker sequence.

**Figure 2:**
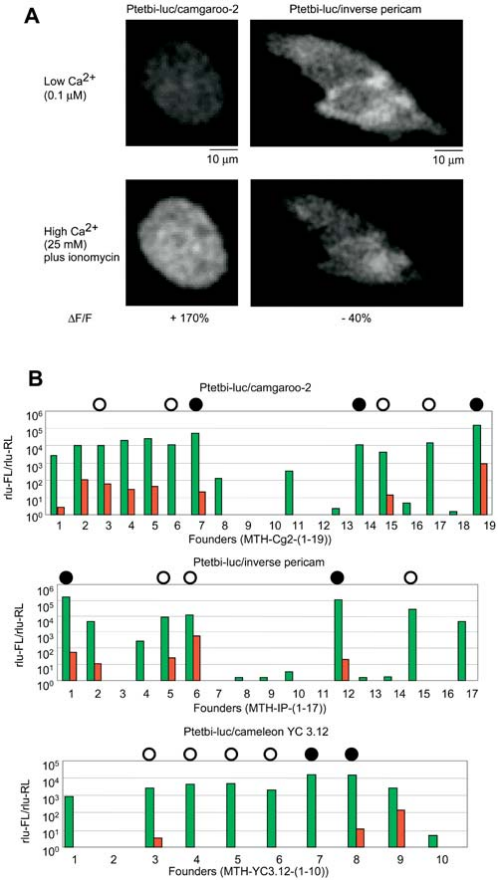
Expression and Functional Tests in Cell Culture (A) HeLa cells expressing tTA and Ptetbi-luciferase/Cg2 or Ptetbi-luiferase/IP and imaged by confocal microscopy. Top row: low [Ca^2+^] (0.1 μM), bottom row: high [Ca^2+^] (25 mM) and ionomycin. Relative fluorescence changes are indicated as %ΔF/F. (B) Ratios (rlu-FL/rlu-RL) of FL to RL activity measured in mouse ear fibroblast cell cultures from all DNA-positive founders in the absence (red) and presence (green) of Dox (see Materials and Methods). Circles (solid and open) indicate the lines that were selected for crossing to the transactivator lines. Solid circles indicate lines that showed smooth fluorescence.

To select indicators for the generation of transgenic mice, we first screened a number of FCIPs in HeLa cells (see Materials and Methods) for brightness, large [Ca^2+^]-depen-dent fluorescence changes, and inducibility. The FCIPs were flash pericam, inverse pericam (IP), G-CaMP, camgaroo-2 (Cg2), and the cameleons YC 2.12 and YC 3.12 (Griesbeck et al. 2001; Nagai et al. 2001, 2002; Nakai et al. 2001). Figure 1A depicts the genetic design of FCIPs. We found relative fluorescence changes (ΔF/F) of approximately +170% and −40% for Cg2 (*n* = 5 cells) and IP (*n* = 2 cells), respectively (Figure 2A), and therefore selected Cg2 and IP for the generation of transgenic animals. In addition, we chose YC3.12, which showed inconclusive results in the screening but is optimized for expression at 37 °C (see Figure 1A). In our rough screen we did not find detectable responses for any of the other indicators.

The TET system (Figure 1B; for a review see Gossen and Bujard 2002) was chosen because it allows combinatorial targeting of different neural cell populations using genetic crosses (Mayford et al. 1996). In addition, we wanted to have temporal control over expression in order to test whether the indicator protein is inactivated by constitutive expression throughout development. The three selected FCIPs (Cg2, IP, and YC3.12), were placed under the control of the bidirectional TET promoter (Ptetbi) (Baron et al. 1995). The opposite side of the Ptetbi contained the firefly luciferase (FL) gene (Baron et al. 1995; Hasan et al. 2001). This allows, using the ear fibroblast method, the screening of founders for the presence of the functional gene without the need for a second generation of crosses into activator lines (Schoenig and Bujard 2003). Of a total of 46 candidate founder animals (Figure 2B), the best four to six founders for each construct (judged by FL/renilla luciferase [RL] luminescence in the fibroblast assay, see Materials and Methods; Figure 2B) were selected for mating with mice expressing the TET-dependent transactivator (tTA) under control of the alpha-calmodulin/calcium-dependent kinase II (αCaMKII) promoter (Mayford et al. 1995, 1996). All selected founders showed, in addition to strong expression, efficient regulation of luciferase activity by doxycycline (Dox) (200- to 20,000-fold increase in luciferase activity with Dox; Figure 2B). All of the following experiments were conducted in the absence of TET derivatives, leaving the controlled genes active.

### Dox Inducibility and Expression Patterns

**Figure 3:**
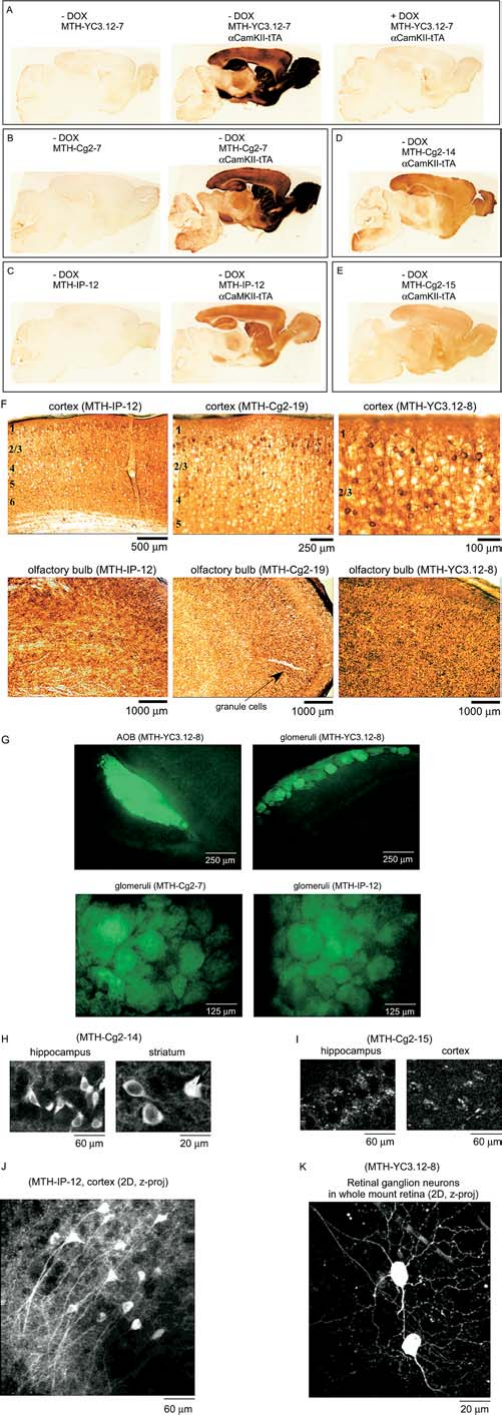
Doxycycline and tTA-Dependent FCIP Expression Immunohistochemical assay (A–F) using rabbit polyclonal GFP antibodies/peroxidase-DAB system: (A) YC3.12, single-positive (MTH-YC3.12-7), double-positive (MTH-YC3.12-7, αCamKII-tTA), and Dox-treated double-positive (MTH-YC3.12-7, αCamKII-tTA). (B) Cg2, single-positive (MTH-Cg2-7) and doubles-positive (MTH-Cg2-7, αCamKII-tTA). (C) IP, single-positive (MTH-IP-12) and double-positive (MTH-IP-12, αCamKII-tTA). (D) Moderate-expression line of Cg2 (MTH-Cg2-14, αCamKII-tTA). (E) Low-expression line (MTH-Cg2-15, αCamKII-tTA). (F) FCIP distribution in various brain areas. (G) Fluorescence in fixed brain slices from the accessory and the main olfactory bulb. (H–K) Two-photon images of acute, living brain slices. (H) Neurons in both CA1 and striatum usually show nuclear exclusion. (I) punctate expression in low-expressing lines (also see Figure 2B, open circles); example from CA1 and cortex. Maximum intensity projection of two-photon 3D stacks taken from a brain slice (J) and a whole-mount retina (K).

Brain slices from double-positive animals (i.e., harboring both *Ptetbi-FCIP/luc* and *αCamKII-tTA* genes) were used for analysis of expression patterns by immunohistochemistry (Figure 3A–3F) and two-photon microscopy (acute brain slices: Figure 3H–3J; whole-mount retina: Figure 3K). Expression of FCIPs was apparent only in double-positive animals and in the absence of Dox (strong to moderate levels in several lines; Figure 3A–3E), and Dox strongly suppressed the expression of FCIPs (Figure 3A). Expression levels varied by line: high in MTH-YC3.12-7, MTH-YC3.12-8, MTH-Cg2-7, MTH-IP-12, and MTH-Cg2-19; moderate in MTH-Cg2-14 and MTH-IP-1; and low in the remaining lines (e.g., Figure 3E; Figure 2B, open circles). FCIP-positive cells included hippocampal and neocortical pyramidal cells and vomeronasal and main olfactory receptor neurons (see Figure 3G for axon fiber projections in the accessory and main olfactory bulb), as well as granule cells and a few mitral cells in the olfactory bulb (Figure 3F and data not shown). Expression of FCIPs was robust in hippocampal areas CA1 and CA3 and in the mossy fiber area of the dentate gyrus (data not shown); cortical and retinal ganglion cell dendrites were clearly identifiable (Figure 3F, 3J, and 3K). The pattern of expression appeared to be a mosaic subset of that of *αCamKII.* There was little obvious variation between lines in gene-expression patterns in most areas except in hippocampal areas CA1 and CA3 and in the dentate gyrus (data not shown). In high- and moderate-expression lines (MTH-Cg2-7, MTH-Cg2-14, MTH-Cg2-19, MTH-IP-1, MTH-IP-12, MTH-YC3.12-7, and MTH-YC3.12-8; closed circles in Figure 2B), consistent with their luciferase activities, cytosolic fluorescence (Figure 3H, 3J, and 3K) was smooth with occasional bright spots, with nuclei usually less fluorescent. Sometimes unusually bright neurons were seen, usually located near the slice surface and presumably damaged. In these cells the nuclei were as bright as or brighter than the cytosol (Figure 3H, arrow). In low-expressing lines (the majority: MTH-Cg2-[3, 6, 15, 17], MTH-IP-[5, 6, 15], and MTH-YC3.12-[3, 4, 5, 6]), fluorescence was punctate (Figure 3I).

**Figure 4:**
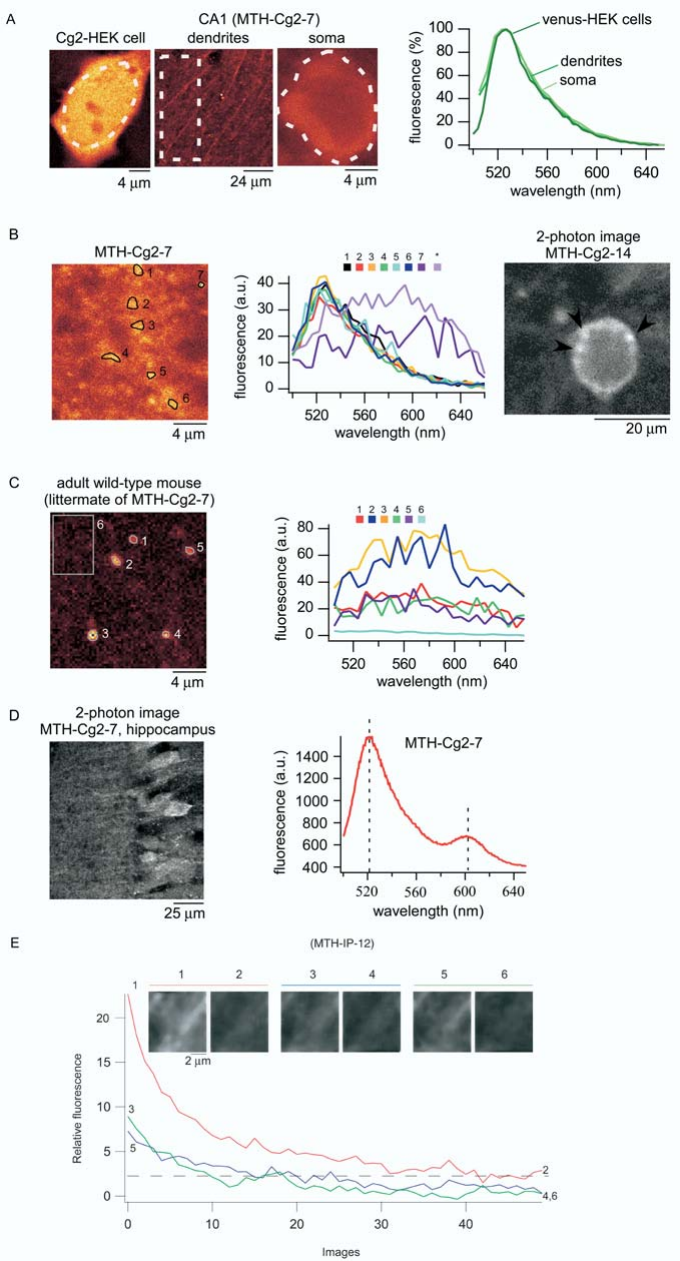
Fluorescence Spectra and FCIP Mobility (A) Fluorescence distribution and emission spectra of Cg2 in cultured HEK cells and in neurons (dendrites and soma) in an acute brain slice (MTH-Cg2-7). (B) Punctate fluorescence and corresponding emission spectra (MTH-Cg2-7). “*” denotes emission spectrum of a punctate fluorescence in a different brain slice (not shown). Note smooth and punctate fluorescence also in the two-photon image on the right (MTH-Cg2-14). (C) Punctate fluorescence in a double-negative littermate of MTH-Cg2-7. (D) Image (left) and emission spectrum (right) of two-photon-excited fluorescence in an acute brain slice (MTH-Cg2-7). (E) Indicator mobility by two-photon fluorescence photobleaching recovery (IP, MTH-IP-12).

We compared the spectral properties of smooth and punctate fluorescence in one high-expression line (MTH-Cg2-7) and one moderate-expression line (MTH-Cg2-14), using confocal imaging spectrometry with excitation at 488 nm. The emission spectra of smooth and punctate fluorescence were similar to each other and to Cg2-expressing HEK cells (Figure 4A and 4B; a two-photon image of punctate fluorescence is shown in Figure 4B, right, arrowheads). Punctate fluorescence was occasionally observed in wild-type and double-negative (−/− *Pbitet-FCIPs* and −/− *αCamKII-tTA*) mice, but there it had very different, much broader emission spectra (Figure 4C). In three of the seven double-positive mice examined, an additional distinct peak was seen at 600 nm, which was never seen in either single-positive or *C57/BL6* wild-type mice (Figure 4D and data not shown). In the further analysis we concentrated mostly on the more promising smooth-fluorescence lines.

To determine what proportion of fluorescent protein is bound or sequestered, and hence immobile, we performed two-photon fluorescence-recovery-after-photobleaching experiments (Svoboda et al. 1996) on both somata and neurites of a high-expressing line (IP, MTH-IP-12) and found that roughly half of the indicator protein is mobile (Figure 4E and data not shown).

Punctate fluorescence and the immobile fraction found in the bleach−recovery experiments suggest that a significant fraction of transgenetically expressed FCIPs interact with other cellular components, possibly via binding of the M13 or calmodulin sequences in FCIPs to their normal cellular targets.

### In Vivo Two-Photon Imaging

**Figure 5:**
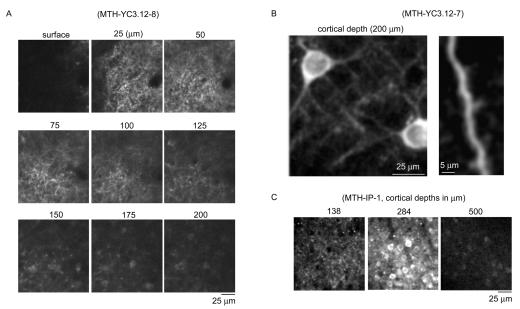
In Vivo Two-Photon Imaging Through the Thinned Skull Yellow cameleon 3.12 at different depths (MTH-YC3.12-8) (A) and with high resolution (MTH-YC3.12-7) (B). (C) IP at different depths (MTH-IP-1).

To evaluate the achievable signal levels in intact animals, we performed in vivo two-photon imaging through the thinned skull in adult anesthetized mice (MTH-YC3.12-8, MTH-YC3.12-7, MTH-IP-1, and MTH-Cg2-7). Imaging up to and occasionally beyond a depth of 500 μm was possible (Figure 5). Densely packed neurites were clearly visible, consistent with the staining patterns seen in acute slices (see Figure 3J) and in histochemical preparations (see Figure 3F). These results show that FCIP fluorescence is sufficiently strong for high resolution in vivo morphological imaging.

### Functional Responses

Next we tested the Ca^2+^ response properties of FCIPs using a variety of different preparations and stimulation methods.

#### Somatic recordings in slices

Mainly to test temporal response characteristics, we performed a series of somatic electrical recording and synaptic stimulation experiments on pyramidal cells in brain slices. Targeted whole-cell tight-seal recordings of FCIP-expressing layer-2/3 cortical cells (the cell identity was confirmed by the overlap of fluorescence from the FCIP and that from Alexa 568, which was contained in the pipette) showed that FCIP-expressing cells have normal electrophysiological properties (Figure 6A). Somatic FCIP fluorescence (recorded with a CCD camera; Figure 6A, left) showed small changes (ΔF/F ∼4%, 10 trials, MTH-Cg2-14; Figure 6A, lower right) in response to trains of current-injection-triggered APs (Figure 6A, upper right). In hippocampal pyramidal cells in area CA1, recorded electrically with sharp high-resistance microelectrodes, two-photon scans of the somatic fluorescence showed larger changes (ΔF/F ∼10%, MTH-Cg2-19; Figure 6B) with a smaller number of APs. The difference in response size may have been due to washout of FCIP into the patch pipette and to the lack of optical sectioning in the CCD measurements, which contributes to an unknown extent to the resting fluorescence from outside the recorded cell. For sharp-electrode recordings, transient fluorescence increases of up to 100% were usually seen during the break in (Cg2, MTH-Cg2-14, and MTH-Cg2-19; data not shown).

**Figure 6:**
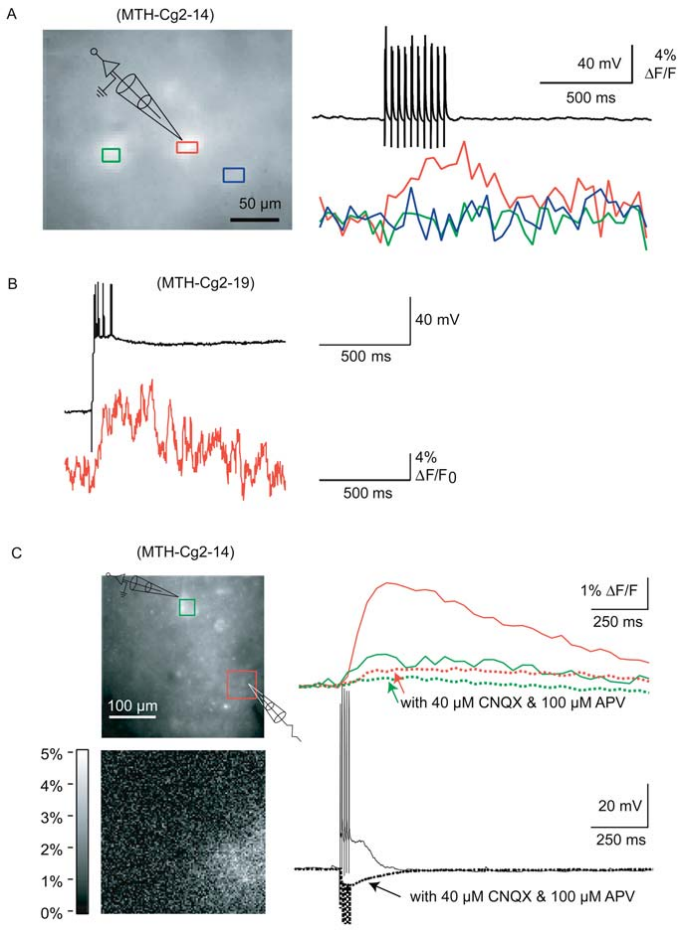
FCIP Responses to Direct and to Synaptic Stimulation in Acute Brain Slices (A) Whole-field imaged responses of Cg2-positive cells in cortex to bursts of APs evoked by somatic current injection (whole-cell recording electrode indicated schematically); responses in the recorded (red) and in a nonrecorded (green) soma and in a region with no cell body (blue). (B) Two-photon line scan (lower trace) through the soma of a hippocampal CA1 pyramidal neuron during a burst of APs evoked by somatic current injection through a high-resistance microelectrode. (C) Whole-field-imaged responses to synaptic stimulation in cortex (five pulses at 100 Hz, 10 μA); ΔF/F image is shown below. Fluorescence and voltage responses with and without pharmacological block of glutamate channels (note suppression of APs and unmasking of inhibitory synaptic potentials).

#### Synaptic stimulation

First, cortical slices from a Cg2 animal (MTH-Cg2-14) were imaged using a CCD camera. Stimulation effectiveness was monitored in a distantly located soma (≈200 μm) by whole-cell tight-seal recording (Figure 6C). Short trains of stimuli (five pulses, 0.1 ms long, at 100 Hz, 10 μA; Figure 6C, lower right) elicited fluorescence increases localized to an area near the stimulating electrode (Figure 6C, upper right). Peak ΔF/F ranged from 3%–8% (15 trials, in three slices). The fluorescence increase began in the frame following the stimulus onset (Figure 6C, right) and was as fast as responses seen in similar experiments with synthetic indicators (Larkum et al. 2003). Smaller fluorescence changes were observed in the soma of the recorded neuron (Figure 6C, upper right, solid green trace). Small changes could also be seen in the neuropil as far as 150 μm from the stimulation site (data not shown). Fluorescence changes were largely abolished by glutamate-receptor blockers 6-cyano-7-nitroquinoxaline-2,3-dione (40 μM) and 2-amino-5-phosphovaleric acid (100 μM) (Figure 6C, upper right, dotted traces), indicating that they were mediated by synaptic activation. Similar experiments were performed using two-photon imaging, which allows optical sectioning and hence better spatial localization and signal-to-noise ratio. Synaptic stimulation again led to reproducible and rapid fluorescence changes. Changes were now much larger for both Cg2 and IP (ΔF/F ∼20%–100%, MTH-Cg2-19, 42 trials from three slices [Figure 6D]; ΔF/F approximately 15%–40%, MTH-Cg2-7, 35 trials from two slices [Figure 6E]; ΔF/F approximately −30%, MTH-IP-12, five trials from one slice [Figure 6F]). Changes were spatially inhomogeneous, showing “hotspot” structures possibly due to the activation of individual synaptic sites (see Figure 6D, lower panel, trace 7; MTH-Cg2-19). Similar results were obtained in dentate gyrus mossy fibers (Figure 6E, note black and blue regions of interest in trace 3). In some experiments response amplitudes started to decrease after a few trials, presumably because of bleaching or photo damage (data not shown).

**Figure 6:**
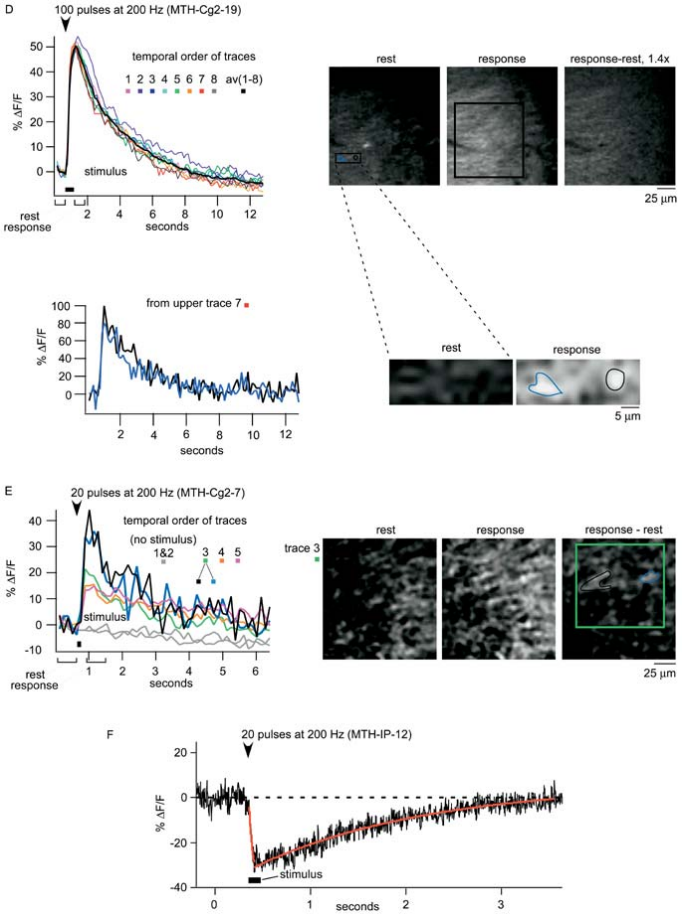
Continued (D–F) Two-photon-imaged responses to synaptic stimulation in the hippocampus. (D) CA1 region with Schaffer collateral stimulation (eight individual response traces and the averaged trace are shown, region of interest indicated in the “response” image). Averaged images (five frames) during rest and response, and their difference, respectively. In localized hot spots, responses reach 100% (panels and traces shown below). (E) Similar response amplitudes and kinetics are seen in the dentate gyrus with mossy fiber stimulation (note that the number of stimuli was only 20). (F) IP responses recorded by a two-photon line scan through a CA1 soma; stimulation (20 pulses at 200 Hz) of neurites (approximately 50 μm away from the somata).

#### Light-evoked responses in retinal whole mount

**Figure 7:**
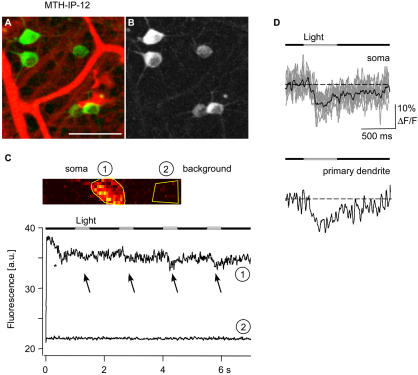
Light-Evoked Ca^2+^ Responses in Retinal Ganglion Cells (A) Intact, light-sensitive retinal whole mount with Sulforhodamine 101 (red) in the extracellular space. Blood vessels are red; IP-positive (MTH-IP-12) retinal ganglion cells are green; and unstained ganglion cells are dark. (Scale bar: 50 μm). (B) Projection of an image stack reveals the IP-labeled primary dendrites of the retinal ganglion cells. (C) Time course of Ca^2+^ response measured by high repetition rate image scan (62.5 Hz) of a soma: The cell responds with a decrease in fluorescence to the onset of the laser (asterisk) and to the repeated light stimulation (arrows). (D) Averaged (four repetitions) light-stimulus-evoked Ca^2+^ response (black trace; gray traces are single trials) measured in the soma (above) and in the primary dendrite (below) of a retinal ganglion cell.

In several mouse lines with YC3.12 (for example MTH-YC3.12-8), a subset of ganglion cells was strongly labeled (see Figure 3K) but no light-induced Ca^2+^ responses were seen (eight cells in two retinas tested), consistent with YC3.12 results in other tissues. In lines expressing Cg2 (MTH-Cg2-14), fluorescence became too weak and bleached too quickly for optophysiological measurements (eight cells in two retinas tested; data not shown). In one of two IP-expressing mice tested (Figure 7A and 7B; MTH-IP-12), the fluorescence levels were high enough to follow axons and primary dendrites. After the onset of laser excitation, the fluorescence in the cells decreased and then stabilized at a slightly lower level (Figure 7C, asterisk). This effect was more pronounced at higher laser power (data not shown) and probably reflects the development of a steady state between photobleaching and diffusional replenishment from outside the excitation volume. Stimulation with spots of visible light evoked transient decreases in fluorescence, i.e., increases in intracellular [Ca^2+^], in both soma and primary dendrites (Figure 7C and 7D). Seven of 12 cells tested in the two IP mice displayed obvious light-evoked somatic Ca^2+^ responses. The variation in response amplitude between cells may in part be due to heterogeneity of the labeled cell population.

#### In vivo imaging of odor responses in the olfactory bulb

**Figure 8:**
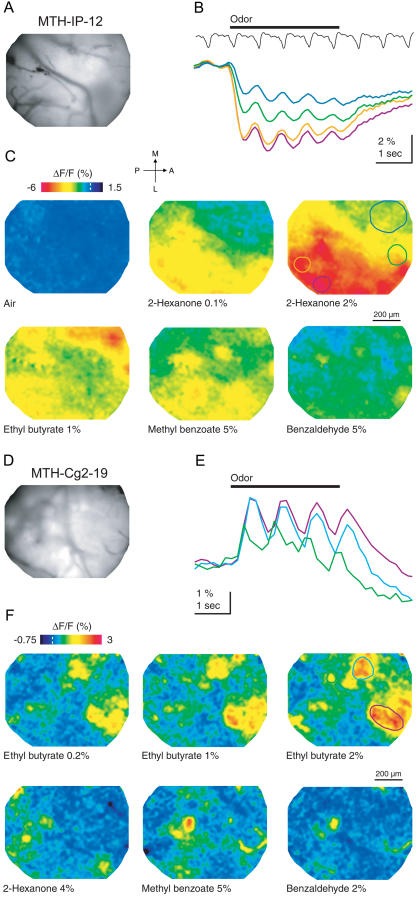
In Vivo Imaging of Odor-Evoked Ca^2+^ Signals with Transgenic Indicators in the Olfactory Bulb (A–C) IP (MTH-IP-12). (A) Raw fluorescence image. (B) Time course of fluorescence signal in the corresponding regions outlined in (C) (matching line colors). The black trace shows respiratory activity. (C) Color-coded map showing the relative change in fluorescence evoked by different odors in each pixel during the first second of the odor response. (D–F) Cg2 (MTH-Cg2-19). (D) Raw fluorescence image. (E) Time course of fluorescence signal in the corresponding regions outlined in (F) (matching line colors). (F) Color-coded maps showing the relative change in fluorescence evoked by different odors in each pixel during the first second of the odor response.

FCIPs are expressed in the olfactory bulb in afferent sensory axons and granule cells, with relative expression levels varying somewhat with FCIP type and across lines (data not shown). Substantial changes in fluorescence were observed for both IP and Cg2 (MTH-IP-12 and MTH-Cg2-19) in response to odor stimulation (Figure 8). We do not know, however, the exact fraction of non-FCIP autofluorescence contained in the resting signal.

Odor-evoked overall fluorescence changes were, as expected, negative in IP animals (MTH-IP-12; Figure 8B, responses seen in 41 of 41 trials) and positive in Cg2 animals (MTH-Cg2-19; Figure 8E, responses seen in 54 of 54 trials). The signals consisted of a sustained component and a periodic component, which was phase-locked to the animal's respiration (Figure 8B and 8E). During some of the late sustained component, the well-known negative intrinsic response (Spors and Grinvald 2002) is likely to be superimposed. Signals were significant even for low odor concentrations (e.g., 0.1% 2-Hexanone; Figure 8C), and they increased with concentration. The largest fluorescence changes seen were −8% for IP and +3% for Cg2. The time course of the signals was consistent with the [Ca^2+^] dynamics in sensory afferents (Wachowiak and Cohen 2001).

Maps of odor-evoked fluorescence changes were constructed during early response times, thereby minimizing the contribution of the slow intrinsic signal. Odor-evoked spatial patterns of Ca^2+^ signals were widespread in MTH-IP-12 (Figure 8C) and more localized in MTH-Cg2-19 (Figure 8F) but in both cases were more diffuse than maps of afferent glomerular activity measured with a synthetic indicator in sensory axon terminals (Wachowiak and Cohen 2001). This is presumably because FCIP is also expressed in granule cells. These receive input from secondary dendrites of mitral cells, which project for several hundreds of micrometers around each glomerulus. Nevertheless, each odor evoked a unique activity map, and odors known to evoke similar maps of glomerular afferent activity (e.g., methyl benzoate and benzaldehyde) evoked similar activity patterns in transgenic animals (Figure 8C and 8F). The more localized signals seen with Cg2 may be due to its lower affinity for Ca^2+^, reporting only high [Ca^2+^] in the vicinity of activated glomeruli. Alternatively, Cg2 might be expressed more strongly in olfactory receptor axons.

## Discussion

We have demonstrated that genetically encoded FCIPs can be stably expressed in mice and are functional. Transgenically expressed FCIPs showed changes of up to 100% in response to synaptic stimulation (see Figure 6D–6F). These changes are smaller than those seen in cell culture or protein extracts (Griesbeck et al. 2001; Nagai et al. 2001), suggesting that a fraction of protein, possibly immobile and sequestered, is nonresponsive. The size of the immobile fraction seen in bleach-recovery experiments (see Figure 4E) fluctuates strongly around a mean value of roughly 50% of the total FCIP fluorescence. Fluorescence changes evoked by electrical stimulation in the neuropil show that FCIPs respond quickly to Ca^2+^ influx. The relative fluorescence changes recorded in slices in response to synaptic stimulation are large when measured by two-photon microscopy (see Figure 6D–6F) but are substantially smaller with wide-field microscopy (see Figure 6C), presumably because signals from activated and nonactivated cells inevitably mix because of a lack of optical sectioning in the wide-field case. In whole-cell tight-seal recordings somatic signals may fade additionally due to washout of responsive protein. Since camgaroos and pericams are intrinsically pH-sensitive (Baird et al. 1999; Griesbeck et al. 2001; Nagai et al. 2001) it is possible that the fluorescence changes contain a component due to changes in [H^+^] (pH) rather than [Ca^2+^]. It is, however, unlikely that the changes we saw are dominated by pH effects for the following reasons. In the case of Cg2, stimulation-induced pH changes (Yu et al. 2003) should lead to a *decrease* in fluorescence while we see an *increase* (see Figure 6D–6F). For IP the change due to pH would be in the same direction, but, in particular, the size of the changes seen during two-photon measurements (see Figure 6F; ∼30%) are almost an order of magnitude larger than what one might expect from pH changes that occur with high [K^+^] stimulation (Yu et al. 2003), but see Wang et al. (1994), who found much larger pH changes, albeit with massive glutamate application. Furthermore, changes of pH are much slower than those seen in our synaptic stimulation experiments.

The robust and fast FCIP signals detected in response to sensory stimulation in vivo confirm that FCIPs are suitable for their main intended use, the imaging of activity from populations of neurons in living animals*.* Crucial for addressing functional questions in neuronal networks will also be the cell-type specificity of expression, which we have demonstrated here for the population of αCamKII-positive neurons.

Our success rate in generating functional transgenic mouse lines for Cg2 and IP was moderate (five of 36 animals that were transgenic, according to DNA typing; see Figure 2B). YC3.12 was a disappointment and did not yield functional lines. This is consistent with a previous attempt to generate transgenic mice expressing YC3.0 under the control of the β-actin promoter (Tsai et al. 2003). There, animals were produced that also showed mosaic expression patterns and had only very small functional signals (ΔR/R ∼1%–2%) when tested by wide-field imaging of cerebellar slices undergoing synaptic stimulation (A. Miyawaki, V. Lev-Ram, and R.Y. Tsien, unpublished data).

An early suggestion that indicator proteins become nonfunctional after expression for an extended period of time (O. Griesbeck, personal communication) certainly does not apply to IP and Cg2 since we found large responses even in mice aged 8–12 wks, which had been expressing indicators since the onset of αCamKII expression before birth. However, even in strongly expressing smooth-fluorescence lines, a substantial fraction of the indicator protein was found to be immobile and potentially nonfunctional at various ages. It is surprising that punctate fluorescence occurs predominantly in weak lines since precipitation typically occurs at high concentrations. It could, however, be that a limited number of binding and sequestration sites for FCIPs exist in the cell and that only after these sites are saturated does the accumulation of mobile (see Figure 3J), cytosolic, and responsive FCIP begin. The accumulation of any functional indicator protein might, therefore, require expression levels above a (rather high) threshold. Such a threshold might explain why in the majority of lines we find weak, nonresponsive, and punctate fluorescence even when there is significant luciferase activity (open circles in Figure 2B). Binding and/or sequestration of FCIP does, of course, raise the specter of interference of the indicator with biochemical processes inside labeled cells. While subtle effects cannot be ruled out at this point, we did not see any obvious abnormalities either at the whole-animal level or at the level of cellular morphology. The labeled neurons, furthermore, are connected synaptically (see Figures 6C, 7, and 8). It will, however, be important to understand and if possible remedy the reasons for the formation of precipitates. Perhaps the use of Ca^2+^-sensing motifs (Heim and Griesbeck 2004) that lack affinity for proteins normally expressed in neurons will eliminate puncta and the immobile fraction. It is unclear why high expression levels appear to be achievable in brain using the TET promoter system but not other, cellular promoters. One explanation for weakness or outright lack of expression might be that sequences within the Ca^2+^-indicator genes silence cellular promoters (Robertson et al. 1995; Clark et al. 1997) but not the tTA-responsive promoters (including Ptetbi). The reason for this, in turn, might be that cellular promoters (unlike the TET promoter) contain a substantial number of transcriptional control elements (upstream activator sequences), any of which might be sensitive to chromatin-induced silencing by the FCIP gene (Lemon and Tjian 2000). This is supported by the observation that attempts to create transgenic mice expressing IP under the control of a 3.5-kb gonadotrophic-releasing-hormone promoter fragment resulted in many lines that had the gene inserted but showed at best weak and punctate expression (D.J. Spergel and P.H. Seeburg, personal communuication), similar to our low-expression lines, while the same promoter fragment drove hGFP2 (an EGFP variant) to high levels (Spergel et al. 1999). Similarly, only weak expression was observed when YC3.1 was placed under control of the neuron-specific enolase promoter (Futatsugi et al. 2002; A. Miyawaki, personal communication).

One of the remaining problems hampering imaging of population activity with FCIPs is that expression, while cell-type specific, is not complete, i.e., not in every cell of one type, even in lines that express FCIPs at high levels. One possible explanation is position effect variegation (PEV), which occurs when a transgene integrates adjacent to a heterochromatin domain in the genome. In such a situation, expression of the locus variegates, being active in some cells and silent in others (Saveliev et al. 2003; Schotta et al. 2003). If this is the case, it might be possible to avoid mosaicism by generating lines free from PEV by cloning FCIP genes into a bacterial artificial chromosome (Shizuya et al. 1992) derived from a TET responder line that is not prone to PEV (Hasan et al. 2001).

In any case, it appears that the use of the TET system allows the expression of genetically encoded Ca^2+^ indicators in mice, albeit for an unexpected reason. Unlike other promoter systems, such as the CMV promoter, which also appears to resist gene silencing, the TET system allows cell specificity via the expression of the transactivator, which appears to be controllable by cellular promoters without gene silencing.

The creation of transgenic mouse lines expressing functional Ca^2+^ indicators opens the way for the measurement of neural activity patterns in mammals in vivo and in vitro. While the sensitivity of genetic indicators does not (yet) quite reach those of synthetic compounds, it is sufficient for single-trial measurements at least in some applications (see Figure 8). Perhaps the greatest advantage is that the genetically encoded indicators alleviate the labeling problem in general and allow the observation of activity in targeted cell populations without the need to load cell or tissue preparations with synthetic indicators using potentially harmful procedures. In vivo imaging of FCIPs will permit analysis of population activity using minimally invasive procedures such as imaging through the intact skull. Applications for FCIPs are similar to those for intrinsic signal imaging, but FCIPs provide substantially higher spatiotemporal resolution and signal-to-noise ratio. Compared to voltage-sensitive dyes, transgenic Ca^2+^ indicators yield substantially larger signals and obviate surgical procedures for dye loading. Another important advantage of transgenic Ca^2+^ indicators is that the optical signal can be interpreted more specifically because of its defined cellular origin. In combination with two-photon microscopy, neuronal activity can be mapped at high resolution, down to the level of individual dendritic branchlets and maybe spines, possibly even in awake, behaving animals (Helmchen et al. 2001). In addition, FCIP lines may be crossed with mouse lines in which the expression of genes of interest has been manipulated. For example, the combination of FCIP mice with lines harboring modifications of plasticity-driven genes (Nakazawa et al. 2002) or genes that cause neurodegenerative diseases (Wong et al. 2002) might help us to understand how specific genes are involved in the construction and experience-dependent modification of brain circuitry.

## Materials and Methods

### 

#### Screening of indicators and generation of transgenic mice

Genes encoding six different Ca^2+^ indicators (flash pericam, IP, CaMP, Cg2, and cameleons YC2.12 and YC3.12) and *FL* were cloned into a Ptetbi vector (Clontech, Palo Alto, California, United States). The resulting plasmids (Ptetbi-FL/FP, Ptetbi-FL/IP, Ptetbi-FL/CaMP, Pbi-FL/Cg2, Ptetbi-FL/YC2.12, and Ptetbi-FL/YC3.12) were sequenced and transfected into HeLa cells that stably express tTA (Gossen and Bujard 1992). Cells were then tested for [Ca^2+^]-dependent fluorescence changes to establish functioning of the indicators (see Figure 1 and Figure 2A). The transgene insert, devoid of vector sequences, was purified by sucrose gradient (Mann and McMahon 1993) and used for the generation of transgenic animals, using the DNA-microinjection method (Gordon and Ruddle 1982) in the facility of the Zentrum für Molekulare Biologie at the University of Heidelberg. All procedures were performed in accordance with German federal guidelines for animal experiments.

#### Screening of founders using cultured ear fibroblasts

Ear fibroblast cultures were prepared for every DNA-positive founder animals using the procedure described by Schoenig and Bujard (2003). Cells were trypsinized after reaching confluency and plated into 6-well plates divided into sets with and without Dox (4-[Dimethylamino]-1,4,4a,5,5a,6,11,12a-octahydro-3,5,10,12,12a-pentahydroxy-6-methyl-1,11-dioxo-2-naphthacenecarboxamide; Sigma-Aldrich, St. Louis, Missouri, United States). When cells reached 50% confluency, both the Dox-plus and the Dox-minus cultures were transfected with 0.5 μg of synthetic reverse tTA (rtTA-M2s) (Gossen et al. 1995; Urlinger et al. 2000) and 0.5 μg of RL plasmids (Promega, Mannheim, Germany) using lipofectamine-2000 DNA-transfection reagents, as recommended by the vendor (Invitrogen Life Technologies, Carlsbad, California, United States). After 48 h, cells were washed once with PBS and incubated in 0.5 ml of lysis buffer on ice (Promega). 50-μl aliquots from each lysate were tested for FL activity and RL activity (Lumat LB9501; Berthold Technologies, Wildbad, Germany). The ratio of FL to RL activity was used to correct for DNA transfection efficiency. Individual transfections and measurements were done in duplicate, usually resulting in normalized activity values that agreed within 5%.

#### Visualizing GFP in fixed brain slices

Brains from double-positive animals (identified by PCR of tail DNA) were fixed in 4% paraformaldehyde in PBS for 4 h and washed twice with PBS. Brain slices were cut to a thickness of approximately 70 μm using a vibratome (VT 1000S; Leica Instruments, Wetzlar, Germany). Distribution of Ca^2+^ indicator protein was determined by staining with GFP-specific polyclonal rabbit antibodies (Clontech) (Krestel et al. 2001) and the DAB peroxidase system (Vectastain ABC Kit; Vector Laboratories, Burlingame, California, United States) or by direct observation of fluorescence with an upright microscope (Zeiss, Oberkochen, Germany) equipped with GFP filters.

#### Two-photon imaging

All two-photon measurements described in the following sections were done using custom-built two-photon microscopes. Fluorescence was two-photon-excited by a mode-locked Ti-sapphire laser (Coherent Mira F900, 930 nm, 100 fs, 78 MHz) coupled into a custom-built imaging system. The objective used was a 40X/0.8 NA water immersion lens (Nikon, Tokyo, Japan). A photomultiplier-based whole-field detector (Denk et al. 1995) detected emitted light in the range around 535 nm, optimized for yellow fluorescent protein signals. Scanning and image acquisition were controlled using custom software (developed by R. Stepnoski, Lucent Technologies, Murray Hill, New Jersey, United States, and M. Muller, Max-Planck Institute for Medical Research, Heidelberg, Germany). Data analysis was performed with ImageJ (http://rsb.info.nih.gov/ij/) and IgorPro (Wavemetrics, Lake Oswego, Oregon, United States).

#### Fluorescence recovery after photobleaching and spectral analyses

Regions rich in neurites were repeatedly scanned in the two-photon microscope. After a first bleaching run (see Figure 4E, red trace, first and last of ten images shown), scanning was interrupted for 15 s. A second bleach run was performed, then scanning was interrupted by 93 s before the final bleach run (see Figure 4E, blue and green traces, pictures 3/4 and 5/6, respectively). Spectral recordings were performed with a confocal microscope (TCS SP2 AOBS; Leica) using an excitation wavelength of 488 nm. Fluorescence emission was measured by recording image sequences with overlapping shifted spectral windows (10 or 20 nm wide) covering the range of 500–650 nm. Spectra were then calculated for different regions of interest and analyzed using the Leica LCS software, Microsoft Excel, and ImageJ.

#### Preparation of living brain slices

Parasagittal and transverse brain slices (300 μm in thickness) from mice (between postnatal days 18 and 60) were prepared according to published procedures for hippocampal experiments (Hoffman et al. 2002) and for cortical experiments (Waters et al. 2003) using a custom-built vibratome (Max Planck Institute, Heidelberg). Mice were deeply anesthetized with halothane. After decapitation the brain was quickly removed and placed into ice-cold, oxygenated artifical cerebrospinal fluid (ACSF; Biometra Biomedizinische Analytik, Gottingen, Germany) containing 125 mM NaCl, 25 mM NaHCO3, 2.5 mM KCl, 1.25 mM NaH2PO4, 1 mM MgCl2, 25 mM glucose, and 2 mM CaCl2 (pH 7.4). For the two-photon imaging experiments, hippocampus slices were incubated at 37 °C for 30 min and then allowed to reach room temperature gradually before being used for experiments over a period of several hours. For the cortical imaging experiments, slices were kept at 34 °C for the duration of the experiment. In all cases the slice chamber was continuously perfused with ACSF.

#### Whole-cell recording and synaptic stimulation in brain slices

Acute brain slices (see above) were maintained in ACSF. Whole-cell tight-seal recordings were made using pipettes made from borosilicate glass (5–10 MΩ) containing 135 mM K gluconate, 4 mM KCl, 10 mM HEPES, 10 mM Na2-phosphocreatine, 4 mM Mg-ATP, and 0.3 mM Na-GTP. Recording pipettes also contained 0.2% biocytin and 1–5 μM Alexa 568, which is spectrally distinct from the FCIPs and can be used to unambiguously identify the recorded cell. For synaptic stimulation we used saline-filled glass electrodes or tungsten microelectrodes (1 MΩ) (World Precision Instruments, Berlin, Germany). Synaptic transmission was blocked in some experiments by the addition of 40 μM 6-cyano-7-nitroquinoxaline-2,3-dione and 100 μM 2-amino-5-phosphovaleric acid.

#### Imaging and analysis in cortical and hippocampal slices

Wide-field images were taken with a MicroMax, 512 × 512 back-illuminated CCD camera (Roper Scientific, Tucson, Arizona, United States) binned 5 by 5. For experiments involving extracellular stimuli we calculated the change in fluorescence relative to the prestimulus period (ΔF/F) for each binned pixel frame by frame. The prestimulus period of regions of interest was fitted with an exponential curve, which was then subtracted from the entire fluorescence time course to correct for bleaching. No corrections were made for autofluorescence, so that the relative FCIP fluorescence changes are likely to be larger. When analyzing action-potential-induced signals from individual neurons, we calculated the fluorescence changes by subtracting the average of two nearby areas from the total fluorescence to account for background fluorescence (the intracellular FCIP concentration may, however, have been reduced by dialysis into the recording pipette—see Discussion).

Two-photon image sequences were collected approximately 50 μm away from the stimulating electrode. For synaptic stimulation five prestimulus frames were collected (64 mm × 64 mm, 128 ms/frame) to record baseline fluorescence (100 pulses at 100 Hz or 20 pulses at 100 Hz). Image sequences were 6 or 12 s long. The background level (average intensity with the laser off) was subtracted from every frame in the image sequence. The average of the five prestimulus (rest) images was subtracted from the average of five “response” images (response minus rest). The built-in smoothing function of ImageJ was used to reduce the noise further. The brightness of the difference image was enhanced for better display. Localized small fluorescent structures or hot spots in neurites were identified visually. For IP, traces with high time resolution were acquired using 64-pixel line scans at 500 Hz. Fluorescence was averaged over the width of the soma. The fluorescence from a neighboring region was subtracted to account for background. Fluorescence changes (percent ΔF/F) were calculated relative to the resting fluorescence.

#### Tissue preparation for light-evoked responses in an intact retinal whole mount

Mice were dark-adapted for several hours before the experiments and all subsequent procedures were carried out under dim red illumination to minimize photobleaching. Animals were anesthetized with halothane and subsequently killed by cerebral dislocation or by intraperitoneal injection of pentobarbital. Immediately afterward, both eyes were removed and dissected free in Ames medium (Sigma-Aldrich). A piece was cut from a retina and placed photoreceptor side down into the recording chamber, and maintained at 35 °C in Ames medium continuously perfused with oxygen. The remaining retina was kept for further use.

#### Ca^2+^ imaging and visual stimulation

The stimulation and imaging procedures were as described elsewhere (Euler et al. 2002). In brief, simple light stimuli (bright spots, 300 μm in diameter, on dark background) were projected repetitively onto the receptive field of a labeled retinal ganglion cell (light spot centered on the cell body) while monitoring Ca^2+^-mediated fluorescence (emission 520 BP 30 nm) changes in retinal ganglion cells using a custom-built two-photon microscope. The laser (Coherent Mira 900F) was tuned to 925 nm (for YC3.12, the laser was tuned to 870 nm, see Results) to keep direct photoreceptor excitation at a minimum and prevent bleaching. To visualize the retinal tissue, Sulforhodamine 101 (2 mg/l; Sigma-Aldrich) was added to the extracellular medium.

#### In vivo imaging in the olfactory bulb

Mice were anesthetized and dissected as previously described (Wachowiak and Cohen 2001). Odors were delivered through a custom-built flow dilution olfactometer. Dilutions are given relative to the stable vapor in the olfactometer's reservoir. Series of images were collected at rates of 5–15 Hz with a cooled CCD camera (CoolSnapHQ; Photometrics, Huntington Beach, California, United States) mounted on a custom-built upright fluorescence microscope that was equipped with a 20×, 0.95 NA water immersion objective (Olympus, Tokyo, Japan) and the following filter sets (Chroma Technology, Rockingham, Vermont, United States): HQ495/30, Q520LP, and HQ545/50 for IP and Cg2, and D436/20, 455DCLP, and D535/30 for YC3.12. For each pixel and frame, the change in fluorescence relative to the pre-odor period (ΔF/F) was calculated. Trials without odor stimulation were subtracted to correct for bleaching. For the display of activity maps, ΔF/F images taken during the first second of odor stimulation were averaged and low-pass spatially filtered. Later times were not included in activity maps to avoid the contribution of intrinsic signals (Spors and Grinvald 2002). Respiratory activity was measured with a piezoelectric strap wrapped around the animal's thorax.

#### In vivo two-photon imaging

Mice were anesthesized with urethane (1.5 mg/g) and body temperature was maintained at 37 °C. For two-photon imaging, a custom-built headplate with an imaging window (4 mm × 3 mm) was glued to the top of the skull using cyano-acrylate (UHU, Buhl-Baden, Germany) and attached to a fixed metal bar before thinning of the skull. The combination of rigid headplate and thinned-skull reduces respiration and cardiac-pulsation-induced brain motion. The microscope objective was positioned at an angle so that the optical axis was perpendicular to the surface of the cortex.

**Table 1:**
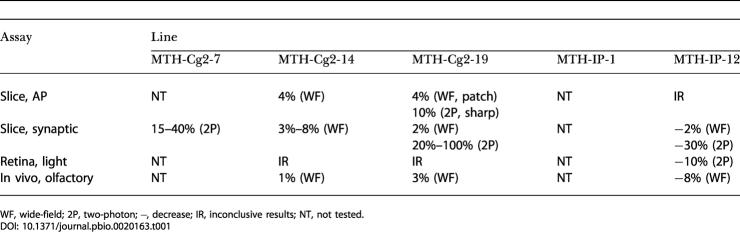
Summary of Functionality Tests Recorded by Either Wide-Field or Two-Photon Imaging

WF, wide-field; 2P, two-photon; −, decrease; IR, inconclusive results; NT, not tested

## Abbreviations

αCamKII: alpha-calmodulin/calcium-dependent kinase II
ΔF/F: relative fluorescence change
AP: action potential
Ca^2+^: calcium
Cg2: camgaroo-2
Dox: doxycycline
FCIP: fluorescent calcium indicator protein
FL: firefly luciferase
GFP: green fluorescent protein
IP: inverse pericam
PEV: position effect variegation
Ptetbi: bidirectional TET promoter
RL: renilla luciferase
TET: tetracycline
tTA: tetracycline-dependent transactivator
